# Effect of Biocontrol Agent *Pseudomonas fluorescens* 2P24 on Soil Fungal Community in Cucumber Rhizosphere Using T-RFLP and DGGE

**DOI:** 10.1371/journal.pone.0031806

**Published:** 2012-02-16

**Authors:** Guanpeng Gao, Danhan Yin, Shengju Chen, Fei Xia, Jie Yang, Qing Li, Wei Wang

**Affiliations:** State Key Laboratory of Bioreactor Engineering, East China University of Science and Technology, Shanghai, China; Argonne National Laboratory, United States of America

## Abstract

Fungi and fungal community play important roles in the soil ecosystem, and the diversity of fungal community could act as natural antagonists of various plant pathogens. Biological control is a promising method to protect plants as chemical pesticides may cause environment pollution. *Pseudomonas fluorescens* 2P24 had strong inhibitory on *Rastonia solanacearum*, *Fusarium oxysporum* and *Rhizoctonia solani*, etc., and was isolated from the wheat rhizosphere take-all decline soils in Shandong province, China. However, its potential effect on soil fungal community was still unknown. In this study, the *gfp*-labeled *P. fluorescens* 2P24 was inoculated into cucumber rhizosphere, and the survival of 2P24 was monitored weekly. The amount decreased from 10^8^ to 10^5^ CFU/g dry soils. The effect of 2P24 on soil fungal community in cucumber rhizosphere was investigated using T-RFLP and DGGE. In T-RFLP analysis, principle component analysis showed that the soil fungal community was greatly influenced at first, digested with restriction enzyme H*inf* I and T*aq* I. However, there was little difference as digested by different enzymes. DGGE results demonstrated that the soil fungal community was greatly shocked at the beginning, but it recovered slowly with the decline of *P. fluorescens* 2P24. Four weeks later, there was little difference between the treatment and control. Generally speaking, the effect of *P. fluorescens* 2P24 on soil fungal community in cucumber rhizosphere was just transient.

## Introduction

Fungi play important roles in soil ecosystem as major decomposers of plant residues, releasing nutrients that sustain and stimulate plant growth in the process [1], [2]. Besides, the phylogenetic diversity of microorganisms can act as natural antagonists of various plant pathogens [3]. A well-developed and diverse rhizosphere community is thought to be critical in the suppression of pathogens [4], [5]. Knowledge of the structure and diversity of the fungal community in the plant rhizosphere will lead to a better understanding of pathogen-antagonist interactions [6].

It is suggested that only 17% of the known fungi can be readily grown in culture [7]. As traditional methods have many pitfalls, culture-independent methods show great potential in monitoring shifts or diversity of microbial community in a variety of environmental samples, such as Phospholipid Fatty Acid analysis (PLFA), Fatty Acid Methyl Ester profile (FAME), Terminal Restriction Fragment Length Polymorphism (T-RFLP), Ribosomal Intergenic Spacer Analysis (RISA), Denaturing/Temperature Gradient Gel Electrophoresis (DGGE/TGGE), Single Strand Configuration Polymorphism (SSCP), Amplified Ribosomal DNA Restriction Analysis (ARDRA), etc. Among these, T-RFLP and DGGE are two most widely used and effective methods in analyzing the spatial and temporal shifts of microbial community. T-RFLP method takes advantage in high throughputs, reproducible and web-based RDP database [8], while DGGE has high resolution by separating the same size fragments and sequencing each band [9]. Thus, in this study, the combination of the two methods would give a better understanding of the soil fungal community in cucumber rhizosphere.

Pesticides are widely used in agriculture to improve the yield of crops. However, chemical pesticides have residues and may influence the ecological system, soil fertility and underground water [10], [11], thus cause seriously environment pollution. Biological control had been a significant approach to plant health management during the twentieth century and promised through modern biotechnology to be even more significant in the twenty-first century [12]. At present, the global markets of biopesticides become larger and larger especially in North America and Europe [13], and the predicted rate of growth is 10% per year [14].

*Pseudomonas* spp. commonly inhabits in soil and has been applied for biocontrol, promoting plant growth and bioremediation. 2, 4- diacetylphloroglucinol(DAPG)-producing strains were major groups in biocontrol microorganisms, because of their easy colonization, good competition and broad antimicrobial spectrum. Thus, they were widely used by more and more researchers [15]–[18]. For example, *P. fluorescens* F113 could inhibit *Erwinia carotovora*, which is the agent of soft rot of potato [19]. It has been also reported that *P. fluorescens* and 2, 4-diacetylphloroglucinol (DAPG) that it produced could prevent *Fusarium oxysporum*, *Septoria tritici*, *Thielaviopsis basicola*, *Rhizoctonia solani* etc [20], [21].

*P. fluorescens* 2P24, which has strong inhibitory on *Rastonia solanacearum*, *F. oxysporum* and *R. solani*, was isolated from the wheat rhizosphere take-all decline soils in Shandong province, China [22]. The root colonization and biocontrol mechanism of it have been studied [23]–[25] and it has been commercialized. However, the potential effect of *P. fluorescens* 2P24 on agricultural soil fungal community is still unknown, as it is important to address the displacements of indigenous microorganisms by inoculates and assess the potential effects on soil microcosm [26].

This was the first study to investigate the effect of *P. fluorescens* 2P24 on soil fungal community in cucumber rhizosphere. Changes in soil fungal community were detected with T-RFLP and DGGE.

## Materials and Methods

### 1 Bacterial strain and inoculation preparation

The *gfp*-labeled *P. fluorescens* 2P24 was cultured on King's medium B (KB) agar plates containing 100 mg of ampicillin and kanamycin liter^−1^. The bacteria were growing in liquid KB medium at 28°C, 150 r/min with 100 mg of ampicillin and kanamycin liter^−1^ for 24 h. Bacterial density was measured as the absorbance of the fermentation broth at 600 nm, with reference to a standard curve calibrated by plate enumeration.

### 2 Experimental site and description

The experimental site was located in the field of National Southern Pesticide Research Centre of Shanghai, China (31.17°N, 121.13°E), where the average annual temperature is 18°C and total rainfall is about 1200 mm per year. Ten plots were established in the experimental area, while each plot contained fifteen cucumber plants. Five plots were treated with *P. fluorescens* 2P24. The fermentation broth of *P. fluorescens* 2P24 was centrifuged to concentrate and then diluted to 2×10^9^ CFU/L by water. And then, 1 liter of these diluents was pooled to the root rhizosphere of each cucumber plant in each plot directly. The other five plots were treated with the same volume water as control.

### 3 Sampling

All soil samples were taken 5–10 cm below the surface and 5 cm away from the plants, the soils were separated by shaking the roots. Soil samples were collected weekly from each treatment and five samples were taken from each plot at each time, mixed and stored at 4°C.

Soil pH and moisture content were immediately determined after sampling. Soil pH was measured using a pH probe and soil moisture was calculated by drying soil at 115°C to a constant dry weight. The soil organic carbon and nitrogen were also measured [27], [28].

The soil fungal quantity was calculated by traditional cultivation method. 1 g of each soil sample was mixed with 99 ml sterile water and then diluted to different concentration gradients. 100 µl of these diluents was cultured on PDA plates with 4 days and counted (each with three replicates).

### 4 Survival of bacterial strain 2P24

Soil samples were dispersed and decimally diluted into sterile water. The dilutions were plated on to KB agar containing 100 mg of ampicillin and kanamycin liter^−1^. The colonies were calculated after culturing for 48 h.

### 5 DNA extraction

750 mg of each soil sample and 1.25 g of silica beads were beaten for 5 min with 3 ml TENP washing buffer (50 mM Tris, 20 mM EDTA, 100 mM NaC1, 1% PVPP, pH 8.5), followed by centrifugation for 5 min. 3 ml SDS, 500 µl lysozyme (20 mg ml^−1^), 500 µl cellulose solution (20 mg ml^−1^) and 15 µl protease K (20 mg ml^−1^) were added and vortexed for 10 min. After incubation at 37°C for 30 min, 125 µl of SDS (20%) and 0.15 g of PVPP were added to the mixture and then incubated at 65°C for 2 h, followed by centrifugation for 10 min (8,000×*g*). The supernatants were transferred to fresh micro-centrifuge tubes and extracted by mixing an equal volume of phenol-chloroform-isoamyl alcohol (25∶24∶1; pH 8.0) followed by centrifugation for 10 min (12,000×*g*). The aqueous phase was removed by addition of an equal volume of chloroform-isoamyl alcohol (24∶1) followed by centrifugation for 10 min (12,000×*g*). Ten percent of total volume of NaAC (3 mol l^−1^, pH 5.2) and sixty percent of total volume of isopropyl alcohol was added, and the total nucleic acids was precipitated at 4°C for 1 h followed by centrifugation for 10 min (12,000×*g*). The final nucleic acids were washed in 70% (v/v) ice-cold ethanol and air dried before re-suspension in 100 µl TE buffer (pH 8.0). At last, DNA solutions were stored at −20°C.

### 6 T-RFLP method

The universal fungal specific primers ITS1-F (5′-CTTGGTCATTTAGAGGAAGTAA-3′) [29] and ITS4 (5′-TCCTCCGCTTATTGATATGC-3′) [30] were used in this study with the forward primer labeled with 6-FAM. PCR was conducted in 25 µl reaction with 12.5 µl Ex Taq (Takara, Japan), 1 µl of extracted DNA, 0.5 µM of each primer and 1% BSA. The thermo cycler reaction conditions were: 5 min initial denaturation at 94°C followed by 35 cycles of 45 s at 94°C, 30 s of annealing at 51°C, and 1 min extension at 72°C. The final extension was 7 min at 72°C. PCR products were purified with PCR purification kits and detected by 1% agarose gel electrophoresis.

Two different restriction enzymes (*Hinf*I, *Taq*I) were used separately. Restriction digests contained 5 U of enzyme, 5 µL of labeled and purified PCR product in a 20-µL total volume. Restrictions were performed with water bath at 37°C for 2 h followed by an inactivation step at 65°C for 15 min.

The samples were separated with GeneScan 1000 Rox (Applied Biosystems) as an internal size standard on an ABI 310 DNA sequencer (Applied Biosystems) using POP6 polymer. Terminal fragments were evaluated by GeneScan Analytical Software.

### 7 DGGE method

NS1 (5′-GTAGTCATATGCTTGTCTC-3′) [30] and GCFung (5′-CGCCCGCCGCGCCCCGCGCCCGGCCCGCCGCCCCCGCCCCATTCCCCGTTACCCGTTG-3′) [31] were chosen for amplification of fungal sequences, which had been proved to be the most suitable for detecting fungal diversities in soil using DGGE analysis [32]. A GC-clamp was added to the terminal primer to improve electrophoretic separation amplicons by DGGE. The PCR reactions were carried out in 50 µl volumes containing 25 µl Ex Taq (TAKARA, Japan), 2 µl of extracted soil DNA and 1.0 µM of each primer, 1% DMSO. The thermo cycling program was: 2 min initial denaturation at 94°C, followed by 35 cycles of 30 s at 94°C, 30 s of annealing at 55°C, and 1 min extension at 72°C. The final extension was 5 min at 72°C. Products were checked by electrophoresis in 1% (w/v) agarose gels and ethidium bromide staining. PCR products from each sample were separated by DGGEK-2401 system (C.B.S. Scientific Company, Inc., USA). The PCR products were separated as follows: 8% polyacrylamide gels and denaturing gradient from 25% to 45% were used; gels were electrophoresed in 1×TAE buffer at 60°C and 80 V for 16 h.

### 8 Data analysis

For T-RFLP analysis, profiles in the range of 50–600 bp were used for principal component analysis [33], which was conducted using the Statistical Product and Service Solutions statistics software 17.0 (SPSS Inc.). Further more, the similarity of different TRF clusters was calculated based on Pearson correlation method by SPSS.

For DGGE analysis, the similarity of cluster analysis was calculated based on the density of different bands in different lane. DGGE banding pattern analysis was conducted to compare by cluster analysis via the underweighted pair group method with mathematical averages (UPGMA), using the VisionWorksLS software (UVP, US).

## Results

### 1 Soil characteristics and culturable fungi

The average pH value of soil samples was 5.0, while the average water content was 19%. This kind of acid soil is very typical in south China. The soil total organic carbon content was about 1.8 g/kg, and the total nitrogen content was about 0.19 g/kg.

**Figure 1:**
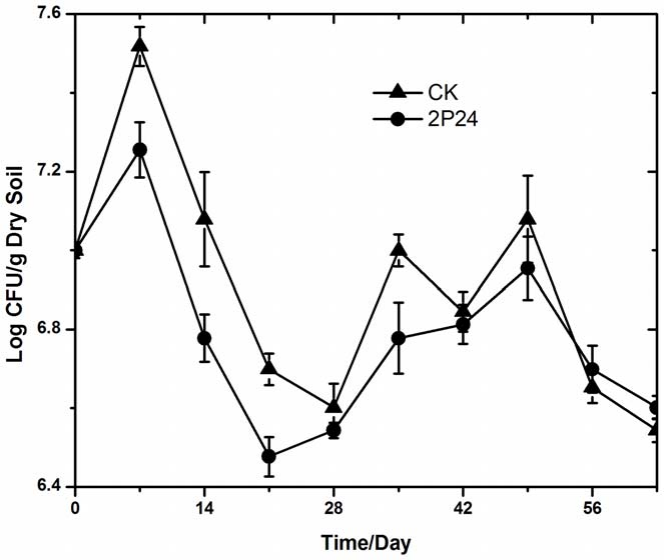
The shifts of soil culturable fungi in cucumber root rhizosphere after inoculation. “CK” was on behalf of the controls amended with water, while “2P24” was on behalf of the treatments amended with *P. fluorescens* 2P24.

As can be seen from Figure 1, the amount of soil culturable fungi in cucumber root rhizosphere decreased after inoculation of *P. fluorescens* 2P24 compared to the controls during the following three weeks. However, the discrimination between the treated and control became to be not very obvious. Through the whole experiment, the amount of soil culturable fungi was about 1×10^7^ CFU/g dry soil on average.

### 2 Survival of *P. fluorescens* 2P24 in cucumber rhizosphere soil

**Figure 2:**
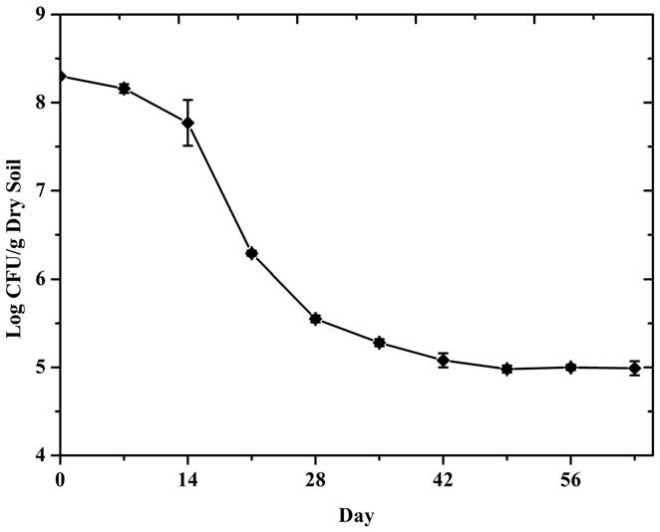
Survival of *P. fluorescens* 2P24 in cucumber rhizosphere soil microcosms after inoculation.

The fermentation broth of *P. fluorescens* 2P24 was inoculated into the cucumber root soil directly. The initial amount of organisms in root soil reached at about 2×10^8^ CFU/g of dry soil. Survival of *P. fluorescens* 2P24 was detected through cultivation method with gradient dilution by distilled water and then calculated after 48 h. During the following days after inoculation, survival of *P. fluorescens* 2P24 decreased sharply (Figure 2). On the 28^th^ day, populations of 2P24 dropped to 3.6×10^6^ CFU/g of dry soil. Then, the survival of 2P24 was not significantly decreased. At the end of this study, the survival of 2P24 still sustained at about 10^5^ CFU/g of dry soil.

### 3 T-RFLP results

T-RFLP was used to detect the fungal community structure in cucumber rhizosphere soil. Although only dominant fungal populations were detected in the T-RFLP method, we assumed that these data represented the total fungal community structure. All of the replicates showed similar results, typical samples were analyzed as follows.

**Figure 3:**
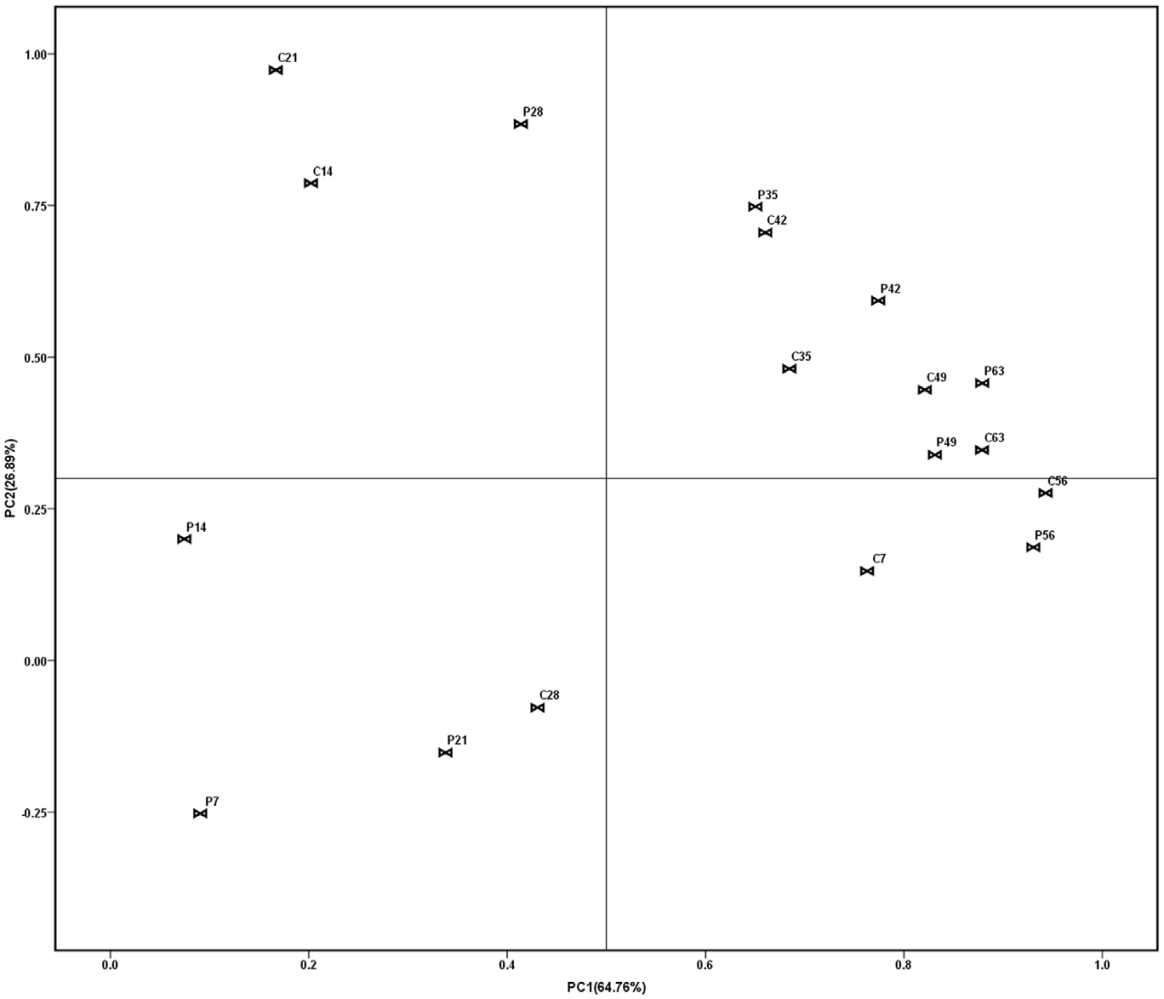
Principal component analysis (PCA) of T-RFLP profiles of soil samples (*Hinf* I). Symbols referred to individual replicates of different treatments. “C” was on behalf of the controls amended with water, and “P” was on behalf of the treatments amended with *P. fluorescens* 2P24. The number (7, 14, 21, 28, 35, 42, 49, 56, 63) following the abbreviation letters “C” and “P” represented the sampling day after inoculation. Numbers in parenthesis were percentage variance explained by each principal component (PC).

**Figure 4:**
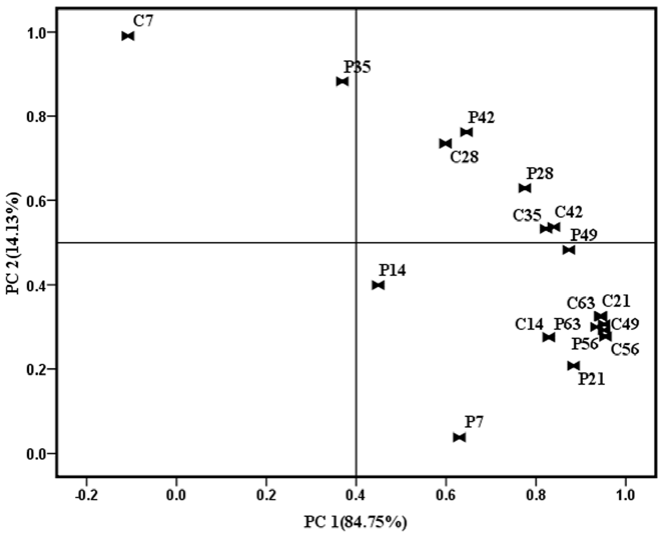
Principal component analysis (PCA) of T-RFLP profiles of soil samples (*Taq* I). Symbols referred to individual replicates of different treatments. “C” was on behalf of the controls amended with water, and “P” was on behalf of the treatments amended with *P. fluorescens* 2P24. The number (7, 14, 21, 28, 35, 42, 49, 56, 63) following the abbreviation letters “C” and “P” represented the sampling day after inoculation. Numbers in parenthesis were percentage variance explained by each principal component (PC).

As can be clearly seen from Figures 3 and 4, a substantial change in the T-RFLP pattern was observed as shown by the Principle Component Analysis (PCA) of the TRF data. However, changes between the control and treatments were significantly different as digested by different enzymes.

Digested by H*inf* I (Figure 3), C7 and P7, C14 and P14, C21 and P21, C28 and P28 were far from each other. P7, P14, P21 and C28 cluster together and they could be regarded as one group. Although C7, C14, C21 and P28 were in the same direction, but there was a long distance from them. It suggested that the soil fungal diversity was greatly changed after the inoculation of 2P24. However, the soil fungal diversity of the treatment became close to the control after four weeks. As can be seen in figure 3, the control and treatment on the 35^th^, 42nd, 49^th^, 56^th^, 63rd day got very close to each other.

Digested by T*aq* I (Figure 4), the soil fungal diversity of control and treatment on the 7^th^ day were totally different. But after one week, the distance between them became shorter and shorter. It indicated that the soil fungal diversity was greatly influenced by the inoculation of 2P24 on the 7^th^ day, and then the soil fungal diversity was gradually recovered.

Even with regard to the soil fungal community of controls, there were some changes as the cucumber grew. Whether digested by H*inf* I or T*aq* I, the controls could not cluster together as one group.

Besides PCA analysis, proximity matrix of different treatments digested by H*inf* I and T*aq* I also showed the similar result (Table 1 and Table 2). For example, in table 1, the correlation coefficient between C7 and P7, C14 and P14, C21 and P21, C28 and P28 was less than 0.8, which meant that these treatments had little relationships. However, the correlation coefficient between control and treatment was between 0.8 and 1, which meant that these treatments had strong relationships. But in table 2, there was no such obvious differences, only the correlation coefficient between C7 and P7 was less than 0.8, while all of the others were more than 0.8, which was similar to the result of PCA analysis.

**Table 1:** Proximity matrix of T-RFLP profiles of soil samples (*Hinf* I).

Proximity Matrix (H*inf* I)																		
	Correlation between Vectors of Values																	
	C7	P7	C14	P14	C21	P21	C28	P28	C35	P35	C42	P42	C48	P48	C56	P56	C63	P63
**C7**	1.000																	
**P7**	.536	1.000																
**C14**	.440	.253	1.000															
**P14**	.154	.180	.704	1.000														
**C21**	.353	.325	.876	.298	1.000													
**P21**	.346	.930	.265	.036	.209	1.000												
**C28**	.245	.898	.168	.010	.116	.992	1.000											
**P28**	.579	.391	.814	.188	.954	.194	.084	1.000										
**C35**	.252	.458	.415	.172	.518	.665	.749	.590	1.000									
**P35**	.536	.047	.658	.128	.815	.207	.318	.910	.860	1.000								
**C42**	.550	.099	.552	.027	.763	.192	.298	.887	.823	.984	1.000							
**P42**	.554	.074	.525	.073	.672	.351	.459	.807	.915	.976	.971	1.000						
**C48**	.469	.276	.410	.091	.522	.537	.635	.660	.967	.909	.896	.973	1.000					
**P48**	.428	.405	.409	.241	.431	.612	.704	.551	.965	.827	.784	.908	.973	1.000				
**C56**	.671	.175	.427	.216	.418	.410	.516	.600	.863	.826	.801	.909	.943	.955	1.000			
**P56**	.580	.313	.358	.247	.321	.522	.618	.490	.875	.759	.722	.861	.930	.971	.987	1.000		
**C63**	.536	.290	.432	.215	.458	.518	.619	.603	.937	.853	.818	.929	.977	.991	.983	.983	1.000	
**P63**	.668	.073	.530	.186	.583	.328	.440	.743	.884	.921	.896	.966	.963	.941	.978	.944	.974	1.000

“C” was on behalf of the controls amended with water, and “P” was on behalf of the treatments amended with *P. fluorescens* 2P24. The number (7, 14, 21, 28, 35, 42, 49, 56, 63) following the abbreviation letters “C” and “P” represented the sampling day after inoculation.

**Table 2:** Proximity matrix of T-RFLP profiles of soil samples (*Taq* I).

Proximity Matrix (T*aq* I)																		
	Correlation between Vectors of Values																	
	C7	P7	C14	P14	C21	P21	C28	P28	C35	P35	C42	P42	C48	P48	C56	P56	C63	P63
**C7**	1.000																	
**P7**	.026	1.000																
**C14**	.221	.906	1.000															
**P14**	.411	.900	.870	1.000														
**C21**	.223	.628	.887	.578	1.000													
**P21**	.144	.879	.993	.815	.915	1.000												
**C28**	.687	.644	.849	.810	.812	.812	1.000											
**P28**	.536	.476	.792	.559	.936	.795	.911	1.000										
**C35**	.422	.410	.742	.436	.944	.761	.833	.983	1.000									
**P35**	.810	.070	.417	.297	.626	.394	.790	.856	.832	1.000								
**C42**	.447	.605	.879	.650	.970	.884	.922	.986	.966	.766	1.000							
**P42**	.686	.448	.752	.606	.857	.735	.954	.980	.938	.911	.954	1.000						
**C48**	.201	.613	.875	.554	.999	.907	.798	.930	.946	.621	.965	.849	1.000					
**P48**	.382	.548	.845	.567	.981	.863	.874	.982	.982	.757	.993	.934	.980	1.000				
**C56**	.176	.635	.887	.578	.993	.922	.793	.911	.925	.584	.955	.830	.996	.972	1.000			
**P56**	.186	.605	.867	.544	.993	.902	.790	.921	.942	.613	.958	.840	.997	.978	.998	1.000		
**C63**	.219	.590	.863	.542	.996	.895	.803	.936	.954	.641	.967	.859	.999	.985	.996	.999	1.000	
**P63**	.184	.469	.777	.413	.976	.823	.735	.921	.959	.655	.936	.833	.983	.971	.978	.987	.988	1.000

“C” was on behalf of the controls amended with water, and “P” was on behalf of the treatments amended with *P. fluorescens* 2P24. The number (7, 14, 21, 28, 35, 42, 49, 56, 63) following the abbreviation letters “C” and “P” represented the sampling day after inoculation.

### 4 DGGE results

**Figure 5:**
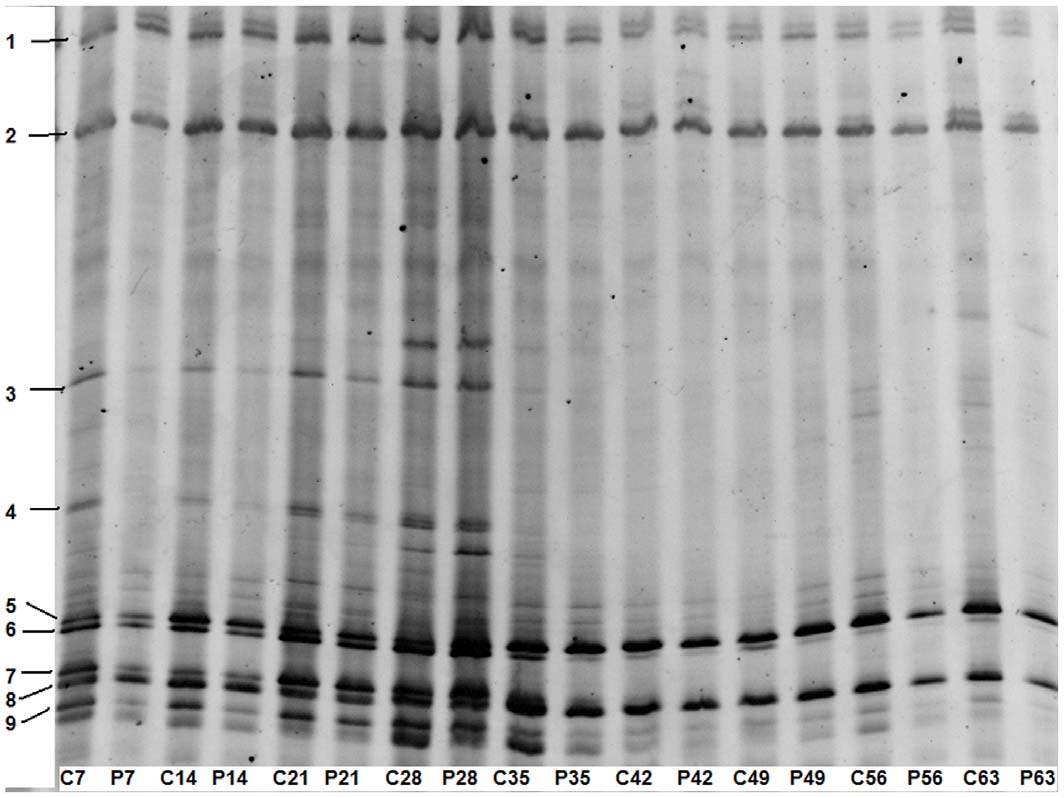
DGGE profiles showed the comparison between the controls and treatments of the soil fungal communities in cucumber rhizosphere after inoculation of *P. fluorescens* 2P24. The fingerprints of fungal communities were generated by separation of 18S rDNA fragments amplified with primers NS1 and GCfung. “C” was on behalf of the controls amended with water, and “P” was on behalf of the treatments amended with *P. fluorescens* 2P24. The number (7, 14, 21, 28, 35, 42, 49, 56, 63) following the abbreviation letters “C” and “P” represented the sampling day after inoculation.

The mixed DNA samples were separated by DGGE fingerprints method. Significant changes between the control and treatment could be observed through the results (Figure 5). All of the replicates showed similar results, typical samples were analyzed as follows.

More than 10 bands could be detected through DGGE analysis. On the 7^th^ day after 2P24 inoculation, bands 3, 4 and 9 of lane P7 almost disappeared compared to lane C7. At the same time, bands 5, 6 and 7 of lane P7 were less concentrated than lane C7. On the 14^th^ day, bands 3, 4 and 9 of lane P14 appeared but were still very dim. Bands 5, 6 and 7 got to be more concentrated than lane P7, but still less than that of lane C14. On the 21^st^ day, the bands of lane P21 came close to the lane C21 except the bands 3 and 4. On the 28^th^ day, the bands of C28 and P28 were mostly similar to each other. After four weeks, the difference between the control and treatment lessened.

**Figure 6:**
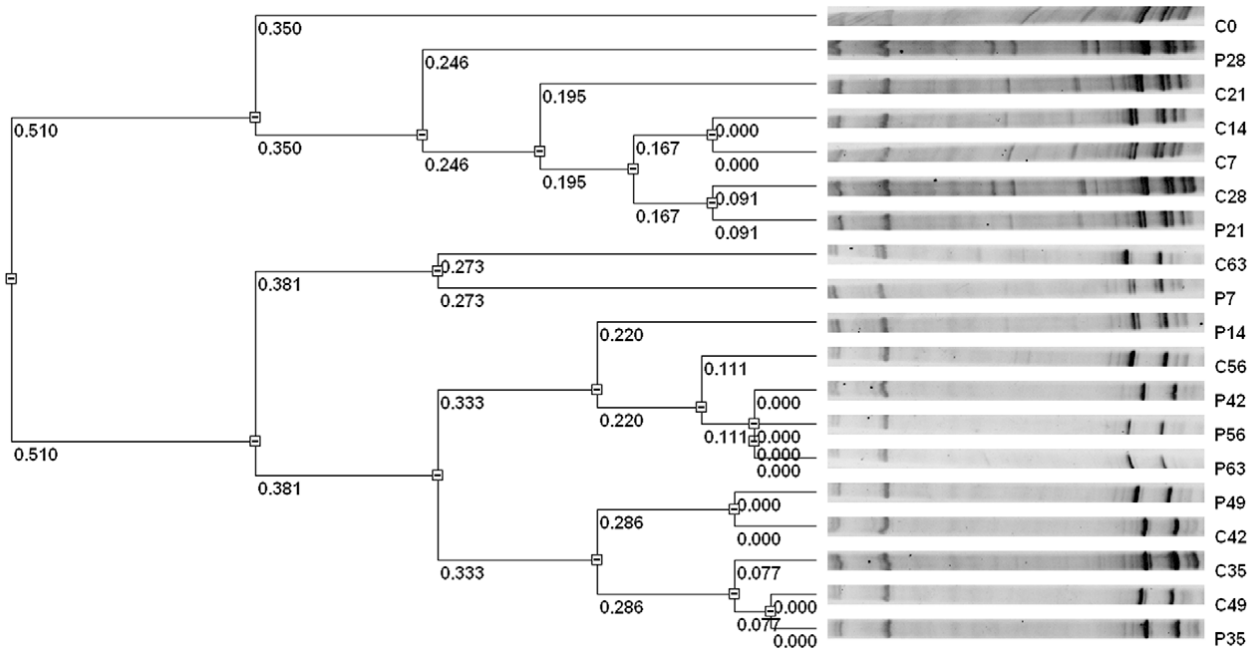
The differences between profiles were indicated by dice similarity. The Dendrogram was based on the RF Values and cluster analysis by the unweighted pair group method analysis (UPGMA) using VisionWorksLS (UVP, US). “C” was on behalf of the controls amended with water, and “P” was on behalf of the treatments amended with *P. fluorescens* 2P24. The number (7, 14, 21, 28, 35, 42, 49, 56, 63) following the abbreviation letters “C” and “P” represented the sampling day after inoculation.

The results of cluster analysis by UPGMA method showed that C0, C7, C14, C21, C28, P21, P28 clustered together as one group while others clustered as one group (Figure 6). C0 was divided as a single branch. P7 and P14 were close to the control and treatment of the following five weeks. There was no big difference between the control and treatment after four weeks.

Overall, the soil fungal community was greatly influenced by the inoculation of 2P24 at first. However, this situation lasted about only two weeks. Four weeks later, the effect of biocontrol agent 2P24 had almost vanished.

## Discussion

Soil microbial community could be affected by various soil conditions, such as pH, moisture, temperature, CO_2_, etc [34]. In this study, the soil was acid soil types, probably because of yearly high temperature and rainfall. However, this kind of soil is a typical agricultural soil both in China and other countries in the world. In general, fungi have been found to be more acid tolerant than bacteria leading to increased fungal dominance in acidic soils [35]–[37]. The soil organic carbon (SOC) and total soil microorganisms mass correlate with the soil water content [38], and it has been proved that 19% of soil water content was most suitable for plant growth and the activities of soil microorganisms [39].

The amount of soil culturable fungi was about 10^7^ CFU/g dry soil. Inoculation of *P. fluorescens* 2P24 decreased the total amount of soil culturable fungi during the following three weeks. It could probably be related with the biocontrol function of *P. fluorescens* 2P24. After that, there was no continued and obvious difference between the treated samples and the controls, which may probably caused by the decreasing of *P. fluorescens* 2P24. Although the amount of total soil fungi was calculated in the experiment, our finally aim was to study the changes of soil fungal diversity, as traditional culture method had a lot of faults.

Many factors could affect the survival of *P. fluorescens* in soil, such as inoculate formulation, soil conditions etc [40], [41]. Thus, *P. fluorescens* would decrease quickly after inoculating into soils, just from 10^7^∼10^9^ to 10^3^∼10^5^ CFU/g dry soil in a month. The difference between variance was mostly dependent on the soil types and initiative inoculation concentrations.

Microorganisms will undergo a large variety of processes following their inoculation, including growth, death, and physiological adaption, conversion to nonculturable cells, physical speed and gene transfer [42]. In the view point of biological invasion, the inoculation of a microorganism may break the original ecological balance of soil microbial community. Our results also showed that the inoculation of *P. fluorescens* 2P24 had a significant effect on the soil fungal community at first. But the effect of *P. fluorescens* 2P24 just lasted about one month, after that, the soil fungal community recovered as the control. Some researchers also found that there were only transient effects on soil microbial communities following the inoculation with biocontrol agents, such as *P.fluorescens* [43], *Streptomyces melanosporofaciens* [44] and *Corynebacterium glutamicum* [45].

In T-RFLP analysis, the great change of soil fungal community could be detected. However, the shift of soil fungal community was different as digested by different enzymes either analyzed by PCA method or by proximity matrix method. The soil fungal community was significantly influenced by the inoculation of biocontrol agent at first. But the process of recovering was totally different as digested by different enzyme. The PCA analysis of data digested by H*inf* I showed that the effect of *P. fluorescens* 2P24 on soil fungal community was very strong until 5 weeks later, while there was slight recovery of soil fungal community by PCA analysis of data digested by T*aq* I.

In DGGE analysis, it could be clearly seen that the soil fungal community recovered little by little after the inoculation of *P. fluorescens* 2P24, and the effect of *P. fluorescens* 2P24 lasted about three weeks. This result was mostly close to the result of T-RFLP analysis digested by T*aq* I.

T-RFLP and DGGE methods have already been applied in analyzing many different environmental samples. T-RFLP takes advantage of analyzing quantitative variances, while DGGE is a better choice to discriminate close species. The combination of these two methods would give a better understanding of soil fungal communities. However, Enwall and Hallin [46] showed that DGGE had a higher resolution than T-RFLP and binary data was better for discriminating between samples. But Smalla *et al* [47] showed that DGGE, T-RFLP, and SSCP analysis led to similar findings, although the fragments amplified comprised different variable regions and lengths. Our findings also showed that DGGE and T-RFLP had similar results, in spite of differences between H*inf* I and T*aq* I in T-RFLP analysis.

As PCR-based methods, T-RFLP and DGGE also have some pitfalls. For example, only dominant species can be amplified from soil DNA. Besides, a lot of factors may affect the final results, such as DNA extraction methods, primers, annealing temperature, Taq polymerase, and restriction enzymes etc [48]. For T-RFLP method, although there are specific RDP database, but identification of a TRF profile is usually impossible especially for fungi. Burke *et al.* [49] approved that T-RFLP could be applied to analyze soil fungi, but it could not reflect the real quantity of soil fungi [50]. Furthermore, two or more species may share the same profile, or one species may distribute in different profiles, and it even outputs pseudo-TRFs [51]. For DGGE method, its fragments were less than 500 bp, which was difficult for the following identification and phylogenetic analysis. Furthermore, sometimes a band did not stand for one species, or one species had different bands just as in T-RFLP method.

### Conclusions

*P. fluorescens* 2P24 is a promising biocontrol strain against many fungal pathogens. However, its impact on soil fungal community is still unknown. This is the first study about monitoring the effect of *P. fluorescens* 2P24 on soil fungal communities in cucumber rhizosphere. After its inoculation, the survival of *P. fluorescens* 2P24 decreased from 10^8^ CFU/g dry soil to 10^5^ CFU/g dry soil during the whole growth time of cucumber. Thus, the soil fungal community was greatly influenced by its inoculation at the beginning. At the same time, the impact of *P. fluorescens* 2P24 on soil fungal community alleviated slowly weekly. Four weeks later, there was little difference between the control and the treatment.

Generally speaking, there was no significant effect of *P. fluorescens* 2P24 on soil fungal community in cucumber rhizosphere in spite of four-week influence. On the contrary, it suggested that the period of validity of biocontrol agent *P. fluorescens* 2P24 may be less than one month. Besides, our study just focused on the whole fungal community, the relationships between *P. fluorescens* 2P24 and each single fungal species was still unknown.
